# Correlation of Antioxidant and Antibacterial Activities of the Aqueous *Pinus pinaster* Aiton Bark Extract Within a Cytocompatible Concentration Range

**DOI:** 10.3390/antiox14111377

**Published:** 2025-11-19

**Authors:** Diana Barros, Liliana Grenho, Maria Helena Fernandes, Pedro Sousa Gomes, Élia Fernandes

**Affiliations:** 1 CISAS-Center for Research and Development in Agrifood Systems and Sustainability, Instituto Politécnico de Viana do Castelo, Rua Escola Industrial e Comercial de Nun’ Álvares, 4900-347 Viana do Castelo, Portugal; eliaf@estg.ipvc.pt; 2 ESTG-IPVC-Escola Superior de Tecnologia e Gestão, Instituto Politécnico de Viana do Castelo, Avenida Atlântico, 644, 4900-348 Viana do Castelo, Portugal; 3 BoneLab, Faculdade de Medicina Dentária, Universidade do Porto, Rua Dr. Manuel Pereira da Silva, 4200-393 Porto, Portugal; lgrenho@fmd.up.pt (L.G.); mhfernandes@fmd.up.pt (M.H.F.); 4 LAQV/REQUIMTE, Faculdade de Medicina Dentária, Universidade do Porto, Rua Dr. Manuel Pereira da Silva, 4200-393 Porto, Portugal

**Keywords:** *Pinus pinaster* Ait. bark, aqueous extract, antioxidant activity, antibacterial activity, *S. aureus*, *E. coli*, cytotoxicity, L929 cells

## Abstract

This study explores the antioxidant, antibacterial, and cytocompatibility properties of aqueous *Pinus pinaster* bark extract (PBE). PBE was prepared using two solvent systems—100% distilled water and 1% DMSO in aqueous solution—at a solid-to-liquid ratio of 1:20 (*w*/*v*), following ISO guidelines. Extract characterization included yield determination, FTIR analysis, quantification of total phenolic (TPC) and flavonoid (TFC) contents, and assessment of antioxidant activity using four complementary methods: free radical scavenging (DPPH and ABTS), metal ion reduction (FRAP), and a competitive reaction assay (ORAC). The phenolic compound profile was further examined by HPLC-DAD. The results indicated that the two extracts exhibited comparable values across all evaluated parameters when expressed per gram of PBE. The TPC and TFC were approximately 400 mg GAE (gallic acid equivalents)/g PBE and 92 mg CE (catechin equivalents)/g PBE, respectively. Antioxidant capacity values were about 880, 1030, 3210, and 585 mg TE (Trolox equivalents)/g PBE for the DPPH, ABTS, ORAC, and FRAP assays, respectively. Furthermore, in both extracts, the phenolic and flavonoid contents exhibited strong positive correlations with antioxidant activity across all four chemical assays. The 100% aqueous extract was additionally evaluated for antibacterial activity and cytocompatibility with eukaryotic cells. Compared to the control, the extract demonstrated IC_50_ values of 0.304, 0.678, and 0.845 mg/mL PBE for the inhibition of *Staphylococcus aureus*, *Escherichia coli*, and fibroblast cells, respectively. Antioxidant and antibacterial activities showed a positive association within concentration ranges that remained non-cytotoxic to fibroblasts. Overall, these findings indicate that the aqueous PBE retains cytocompatibility across a wide concentration range while maintaining both antioxidant and antibacterial activities, underscoring its potential for biological applications involving direct contact with eukaryotic cells.

## 1. Introduction

Maritime pine forests (*Pinus pinaster* Aiton) account for approximately 23% of Portugal’s forested areas [1]. Each year, significant quantities of pine waste are generated [2], prompting interest in utilizing wild pine bark—a byproduct of the lumber industry—as a renewable and sustainable source with large amounts of polyphenols that represent the main group of secondary metabolites in plants [3,4]. The extraction of bioactive compounds from maritime pine biomass has gained considerable attention due to its potential to create value across diverse applications [5,6]. Procyanidins, flavonoids, and phenolic acids are among the concentrated forms of phenolic chemicals found in bark extracts [7], with antioxidant and antimicrobial properties [6]. Maritime pine extracts hold significant promise for therapeutic uses, food enrichment, and biomaterial development [8,9]. Pharmacokinetic studies based on plasma concentration profiles have shown that low-molecular-weight constituents of pine bark extracts, such as catechin, caffeic acid, ferulic acid, and taxifolin, are readily absorbed from the small intestine into the systemic circulation [7]. Consequently, numerous clinical studies have investigated pine bark extracts for various health conditions, with the majority focusing on their effects on cardiovascular health [10]. In this context, a recent systematic review and meta-analysis of randomized controlled trials highlighted that pine bark extracts exert significant effects in humans, including reductions in systolic and diastolic blood pressure, blood glucose levels, and LDL cholesterol, suggesting their potential role in the management and prevention of cardiovascular disorders [11].

Growing public awareness is driving the quest for alternatives for currently undervalued forest leftovers and the application of alternative methods for the sustainable extraction of value-added substances found in these matrices. In order to improve efficiency and extract quality while reducing extraction time and solvent use, “greener” systems—such as innovative extraction techniques—have been used [12]. Further, water extraction has been employed to obtain polyphenol-rich extracts from various pine species, as a sustainable and “green” process [13]. Raising the temperature can facilitate extraction by improving the solubility of phenolic compounds in water. It may also accelerate the extraction process, thereby reducing the extraction time [13].

The bark of many pine species has been extracted with water to create extracts rich in polyphenols for industrial usage [13]. Presently, the products are marketed globally under the names Pycnogenol^®^ (Horphag Research, Geneva, Switzerland), and Flavangenol^®^ (Toyo Shinyaku Inc., Tosu City, Saga, Japan) [14,15,16], Oligopin^®^ (DRT, Firmenich Group, Dax, France) [17] and recently Cosmythic^®^ (Purextract, Firmenich Group, Dax, France), all referring their Maritime pine bark origin to the Landes forest in France, and Enzogenol^®^ (Enzo Nutraceuticals Ltd., Paeroa, New Zealand) from *P. radiata* bark. These formulations are advised to be used for anti-aging due to a variety of effects. The antioxidant properties of pine bark extracts are believed to explain many of their clinical effects [16].

In a previous study [18], we analysed the chemical composition of the bark from a certified *Pinus pinaster* Aiton subsp. *atlantica* plantation located in Portugal’s Minho region, identifying it as a promising source of bioactive compounds. Building on these findings, the present work focuses on preparing aqueous bark extracts using microwave-assisted extraction, with 100% water and 1% DMSO as solvents. The extracts were comprehensively analyzed by assessing extraction yield, polyphenol presence (via FTIR), total phenolic and flavonoid contents, and antioxidant capacity through four complementary assays: scavenging of stable free radicals (DPPH, ABTS), metal ion reduction (FRAP), and oxygen radical absorbance capacity (ORAC). In addition, the phenolic profile was determined by HPLC-DAD. Under the same extraction conditions, the 100% water extract—selected as the greenest solvent system—was also tested for antibacterial activity against *Staphylococcus aureus* and *Escherichia coli*, as well as for cytotoxicity/cytocompatibility in L929 fibroblast cultures. The overall aim was to integrate antioxidant, antibacterial, and cytocompatibility data to validate the efficacy and biosafety of aqueous *P. pinaster* bark extract as a polyphenol-rich plant material suitable for interactions with living eukaryotic cells.

## 2. Materials and Methods

### 2.1. Materials and Reagents

The following reagents were used for the preparation and characterization of *Pinus pinaster* bark and *Pine pinaster* bark extracts: gallic acid monohydrate (Acros Organics, Geel, Belgium), Folin–Ciocalteu reagent and aluminum chloride (AlCl_3_) (PanReac, Darmstadt, Germany), anhydrous sodium carbonate (PanReac, Barcelona, Spain), methanol (Fisher Scientific, Leicestershire, UK), ABTS (2,2′-azino-bis(3-ethylbenzothiazoline-6-sulfonic acid)), cinnamic acid, quercetin, syringic acid, caffeic acid, taxifolin, ferulic acid, ellagic acid, protocatechuic acid, gallocatechin, vanillin, resveratrol, o-coumaric acid, (−)epicatechin, (+)catechin, TPTZ (2,4,6-Tris(2-pyridyl)-s-triazine) and dimethyl sulfoxide (DMSO, ≥99.9%) (Sigma Aldrich, St. Louis, MO, USA), DPPH (2,2-diphenyl-1-picrylhydrazyl) and AAPH (2′-Azobis (2-methylpropionamidine) dihydrochloride) (Sigma Aldrich, Steinheim, Germany), and Trolox (6-hydroxy-2,5,7,8-tetramethylchroman-2-carboxylic acid) (Sigma Aldrich, Buchs, Switzerland). Reagents used for the biological profile of the aqueous *Pinus pinaster* extract included: tryptic soy broth (TSB, Liofilchem, Italy), resazurin solution and MTT solution (Sigma-Aldrich, St. Louis, MO, USA), alpha-minimum essential medium (α-MEM), fetal bovine serum (FBS), Antibiotic-Antimycotic solution (Anti-Anti) (the three reagents from Gibco, New York, NY, USA), Calcein AM (BioLegend, San Diego, CA, USA), and propidium iodide (PI, BD Biosciences, Milpitas, CA, USA). The gallic acid monohydrate, cinnamic acid, quercetin, syringic acid, caffeic acid, taxifolin, ferulic acid, ellagic acid, protocatechuic acid, gallocatechin, vanillin, resveratrol, o-coumaric acid, (−)epicatechin, (+)catechin were HPLC grade. The other chemicals and solvents were analytical grade, and water was obtained by deionization after distillation.

### 2.2. Plant Material—Pinus pinaster Bark

#### 2.2.1. Sample Preparation and Characterization

In Valença, northern Portugal, pine bark from *Pinus pinaster* Aiton subsp. *atlantica* was collected during spring 2021. The trees were part of an experimental and certified plantation located in the forested Minho region (localized around the latitude 41.98 and longitude −8.67) managed by Centro PINUS—Associação para a Valorização da Floresta de Pinho (https://www.centropinus.org/pages/o-que-nos-move (accessed on 18 June 2025)). The pine bark was obtained from 21-year-old trees, and samples were collected by making a circular incision in the main trunk of freshly felled trees, approximately 130 cm above the ground. The species identification was performed based on morphological characteristics consistent with *Pinus pinaster* Aiton, verified by specialists associated with Centro PINUS.

The collected pine bark was thoroughly washed with distilled water several times to remove dirt, lichens, and resin. It was then dried at 40 °C for 48 h, milled, and sieved using an amplitude of 0.2 mm for 1 min (Analysette 3 PRO, Fritsch, Idar-Oberstein, Germany) to select particles with diameters ranging from 200 to 850 μm. Finally, the processed pine bark (PB) was stored in sealed bags in a dry, dark environment until further analysis.

#### 2.2.2. Granulometry

The particle size distribution was determined using a laser diffraction particle sizer, Mastersizer Hydro 3000 (Malvern Panalytical, Worcestershire, UK), equipped with a sample dispersion unit, Hydro LV, of 600 mL volume capacity and an incident laser beam passing through the dispersed particulate sample.

#### 2.2.3. Chemical Analyses

Pine bark was analyzed for moisture, ash, protein, and fat contents using AOAC (1995) [19] procedures, methods AOAC 930.04, AOAC 930.05, AOAC 978.04, and AOAC 920.39, respectively. The determination of extractives using the solvents toluene–ethanol (T:E), ethanol (E), and deionized water (W) was performed according to the ASTM D 1105-96 method (2001) [20]. The crude cellulose, α-cellulose, hemicelluloses, and lignin (acid-soluble and acid-insoluble lignin) contents were determined according to Barros et al. (2023) [18] and Ona et al. (1995) [21]. The lignin procedure used a two-step acid hydrolysis to fractionate the biomass, followed by vacuum filtration. Acid-soluble lignin was measured in the filtrate, and acid-insoluble lignin in the filtration solid fraction. The crude cellulose determination involved the sample reflux with a mixture of acetic acid and nitric acid, followed by vacuum filtration. To quantify holocellulose, the sample was delignified with the addition of sodium hypochlorite in acidic conditions at a temperature of 60 °C. The α-Cellulose was obtained by subjecting a holocellulose sample to alkaline treatment followed by acid treatment. Hemicellulose was calculated by subtracting α-cellulose from holocellulose. The lignin and holocellulose determinations were conducted in the sample after removing extractives. Mineral analysis was performed with the ash from the sample hot-dissolved in nitric acid. Phosphorus content was obtained by molecular absorption spectrophotometry (MAS; Hitachi; U 1100 Spectrophotometer, Tokyo, Japan) using the ascorbic acid method (SMEWW 4500-P E) [22], and the other mineral concentrations were obtained by atomic absorption spectrometry with an air–acetylene flame (Varian AA300, Mulgrave, Victoria, Australia) (SMEWW 3111 B) [22]. The sample used for the above determinations corresponded to the ground crude bark, as described in Section 2.2.1.

### 2.3. Pine pinaster Bark Extracts

#### 2.3.1. Microwave-Assisted Extraction (MAE) Processes

Pine bark extract (PBE) was obtained by microwave-assisted extraction (ETHOS X microwave extraction system with SK-12 medium pressure rotor, Milestone, Italy). The extraction process lasted 15 min under microwave irradiation at 1600 W and a temperature of 130 °C, with a sample-solvent ratio of 1:20 (*w*/*v*) (Barros et al., 2025) [23]. Water and a 1% DMSO aqueous solution were used as extraction solvents.

Closed-system microwave-assisted extraction (MAE) represents a valuable and efficient technique for the recovery of bioactive compounds from lignocellulosic materials. The efficiency of MAE can be influenced by multiple operational parameters, including microwave frequency and power, irradiation time, moisture content, particle size, solid-to-liquid ratio, solvent type and composition, temperature, pressure, and the number of extraction cycles [24]. This method offers significant advantages over conventional extraction techniques, particularly in terms of reduced processing time, lower energy consumption, and improved environmental compatibility [6,25]. Moreover, microwave heating has been demonstrated to be highly effective for the extraction of various classes of compounds, such as alkaloids, terpenes, polynuclear aromatic hydrocarbons, and phenolic compounds [24].

Dimethyl sulfoxide (DMSO) was added to the extraction solvent (water) due to its ability to dissolve a wide variety of polar and nonpolar compounds, low toxicity, high stability of the extracted compounds, and compatibility with biological systems [26]. Although bioactive polar flavonoids, semipolar bioactive flavonoids, and terpenoids can be extracted using polar organic solvents (e.g., methanol, acetonitrile, or cosolvents), these solvents were not considered in this study because they have disadvantages, including toxicity, low vapor pressures, flammability, or pollution, making them ineligible for the application of their extracts in the food, pharmaceutical, or cosmetic industries [27]. The 1% DMSO concentration used is consistent with the study by Selvakumar and Sivashanmugam (2020) [28], who obtained the highest extract yield and total phenolic content with an aqueous solvent of 0.9% DMSO and apparently did not affect cell viability [26]. These characteristics seem to indicate the possibility of efficient extraction of components, including phenolic compounds, from various sample types.

#### 2.3.2. Extraction Yield

The extraction yield reflects the efficiency of a solvent in extracting specific components from the original material. It is defined as the mass of solid extract recovered relative to the initial mass of dry bark [29], being expressed as a percentage (% *w*/*w*). The extraction yield was determined by extract drying at 105 ± 5 °C [19]. The yield of antioxidant compounds extracted from plant material is largely influenced by the conditions under which the liquid–solid extraction process is conducted [30].

#### 2.3.3. Fourier Transform Infrared Spectroscopy

The lyophilized extract of pine bark (PBE) was subjected to Fourier Transform Infrared Spectroscopy (FTIR) to assess the existence of polyphenols. The analysis of the chemical groups and bonding configurations present in the extract was performed by a Thermo Scientific Infrared Spectrometer (Nicolet iS5 FTIR, Waltham, MA, USA) equipped with an attenuated total reflectance (ATR) cell composed of diamond. FTIR spectra were generated at 4 cm^−1^ resolution, comprising a total of 32 scans and covering the frequency range from 4000 to 500 cm^−1^.

#### 2.3.4. Total Polyphenols and Flavonoids Contents, and Antioxidant Activity

The total phenolic compound (TPC) content was measured using molecular absorption spectrophotometry at 725 nm (UV-Vis scanning spectrophotometer VWR^®^—UV-3100PC, VWR^®^, Radnor, PA, USA) following the Folin–Ciocalteu method described by Singleton and Rossi (1965) [31]. Gallic acid was used as the calibration standard (R^2^ = 0.9988), and the results were expressed as milligrams of gallic acid equivalents (GAEs) per mL of extract (mg GAE/mL PBE), per gram of extract (mg GAE/g PBE), and per gram of pine bark (mg GAE/g PB).

The total flavonoid content (TFC) was determined using the method described by Barros et al. (2010) [32]. The method uses molecular absorption spectrophotometry, with readings taken at 510 nm. The standard curve was constructed using (+)-catechin (R^2^ = 0.9999), and the results were expressed as milligrams of catechin equivalents (CEs) per mL of extract (mg CE/mL PBE), per gram of extract (mg CE/g PBE), and per gram of pine bark (mg CE/g PB).

The antioxidant activity was evaluated by the DPPH, ABTS, ORAC, and FRAP assays.

The anti-radical capacity of the samples was determined by DPPH (2,2-diphenyl-1-picrylhydrazyl) and ABTS (2,2′-azino-bis(3-ethylbenzothiazoline-6-sulfonic acid) methodologies, according to Lee et al. (2019) [33]. The mechanism of the free radical scavenging (DPPH) assay consists of the reduction reaction of the DPPH• radical to non-radical DPPH-H in the presence of hydrogen-donating antioxidants, with a color change from purple to yellow and a reduction in absorbance at 517 nm. The purple color discoloration acts as an indicator of antioxidant activity, and the absorbance value measured reflects the antioxidant capacity. The ABTS scavenging activity test measures the capacity of antioxidants to neutralize the stable radical cation 2,2′-azino-bis(3-ethylbenzothiazoline-6-sulfonic acid) (ABTS+), a blue-green chromophore with maximum absorption at 734 nm that decreases in intensity in the presence of antioxidants.

The ORAC (oxygen radical absorbance capacity) test evaluates the antioxidant effect by interrupting the free radical chain reaction, by inhibiting the hydrogen transfer reaction. The test uses 2,2-azobis(2-amidopropane) dihydrochloride (AAPH) as the peroxyl radical source and fluorescein as the fluorescent indicator [34]. The antioxidant activity by the ORAC test was performed according to the method described by Coscueta et al. (2020) [35].

The Ferric reducing antioxidant power (FRAP assay), performed according to Rothe et al. (2023) [36], is based on the oxidation-reduction reaction between [Fe^3+^-(TPTZ)_2_]^3+^ (tripyridyl triazine-ferric complex) and antioxidants (detecting the antioxidants electron donor capacity), that is, the capacity of the antioxidants to reduce [Fe^3+^-(TPTZ)_2_]^3+^ to [Fe^2+^-(TPTZ)_2_]^2+^ (ferric ion to ferrous ion: Fe^3+^ → Fe^2+^), which presents a blue color and has strong absorption at 593 nm [34].

The standard used in the different assays calibration curves was Trolox (DPPH: R^2^ = 0.9991; ABTS: R^2^ = 0.9984; ORAC: R^2^ = 0.9919; FRAP: R^2^ = 0.9998), with the results expressed in milligrams of Trolox equivalents (TEs) per mL of extract (mg TE/mL PBE), per gram of extract (mg TE/g PBE), and per gram of pine bark (mg TE/g PB). In the FRAP assay, iron (II) sulphate was also used as the calibration curve standard (FRAP: R^2^ = 1), with the results expressed in milligrams equivalent (Fe^2+^) per mL of extract (mg Fe^2+^/mL PBE), per gram of extract (mg Fe^2+^/g PBE), and also per gram of pine bark (mg Fe^2+^/g PB), or mM of ferrous ion equivalent (mM Fe^2+^ in the extract).

The smallest amount of extract used in the determination of TPC, TFC, DPPH, ABTS, and FRAP was the result of the 1/250 dilution of the original extracts. For the ORAC test, the smallest amount used resulted from a 1/1000 dilution of the extracts initially obtained. The antioxidant tests were performed in a black 96-well microplate (Thermo Scientific™ Nunc MicroWell). The results expressed in mass were calculated based on the dry matter (DM) of both the extracts (PBEs) and the pine bark (PB).

#### 2.3.5. Extracts’ Phenolic Compound Profile Using HPLC-DAD Analysis

The phenolic compound concentration of pine bark extracts was analyzed on an HPLC system (Thermo Scientific UltiMate 3000, Thermo Fisher Scientific, Waltham, MA, USA) equipped with a Diode Array Detector (DAD). Separation was performed with a Hypersil ODS C18 250 × 4.6 mm 5 µm column (Thermo Scientific, Waltham, MA, USA) at a temperature of 40 °C. The mobile phase was water/formic acid (0.1%) (channel A) and acetonitrile (channel B), with an elution gradient of 1 mL/min for 16 min. Throughout the analysis, the channel B eluent’s mobile phase composition was 15% from 0 to 1 min, an increase to 35% until 9 min, and 90% up to 14 min, followed by a decrease to 15% during 2 min. Phenolic compounds were identified at wavelengths of 280 and 320 nm, based on retention times obtained in the UV spectra for the different standards [23]. Calibration curves in methanol were carried out for low (0.050 to 5.0 mg/L) and high concentration (0.50 to 300 mg/L) ranges for each compound, and the detection limits (LoD) were calculated by the low concentration range calibration curves (Table 1). For injection, extracts containing 10 mg/mL in methanol/water (50:50, *v*/*v*) were filtered through a 0.2 µm PVDF syringe filter. Standard solutions and pine bark extracts were analyzed in triplicate, using a 20 µL injection volume.

### 2.4. Biological Profile of Pine Bark Extracts

#### 2.4.1. Antibacterial Susceptibility Testing

The antibacterial activity of the 100% water Pine bark extract was evaluated against *Staphylococcus aureus* ATCC 25,923 (Gram-positive) and *Escherichia coli* ATCC 25,922 (Gram-negative). Bacterial suspensions were prepared in TSB and grown to the exponential phase before being adjusted to ca. 10^6^ colony-forming units (CFUs)/mL. Pine bark extract dilutions were prepared in TSB at final concentrations ranging from 0.0029 to 1.450 mg PBE/mL and dispensed into 96-well plates. The bacterial suspensions were then added to each well. Control wells contained bacteria cultured in TSB without Pine bark extract. After incubation at 37 °C for 24 h, 10% resazurin solution (0.1 mg/mL) was added to all wells, followed by further incubation. Color changes from purple to pink indicated bacterial growth, which was visually assessed. Fluorescence intensity was measured at 530 nm excitation and 590 nm emission wavelengths using a microplate reader (Synergy HT, BioTek, Winooski, VT 05404-0998, USA). The viability of control bacteria was set at 100%, and the relative viability of treated groups was calculated accordingly.

#### 2.4.2. Cell Culture and Cytotoxicity Assay

The fibroblast cell line L929 (NCTC clone 929, ATCC) was used to assess the cytotoxicity of the 100% water Pine bark extract. Cells were cultured in α-MEM supplemented with 10% FBS and 1% Anti-Anti solution at 37 °C in a humidified atmosphere with 5% CO_2_. L929 cells were seeded at a density of 10^4^/cm^2^ in 96-well plates and allowed to adhere for 24 h before treatment. Pine bark extract dilutions, prepared in α-MEM at concentrations ranging from 0.0029 to 1.450 mg PBE/mL, were then added. Control wells contained untreated cells (Pine bark extract-free). Cell viability and metabolic activity were evaluated over 3 days.

Live/Dead Staining. Cell viability was assessed using Calcein AM and PI staining. After 24 h of exposure to Pine bark extract, the culture medium was removed, and cells were incubated in the dark for 30 min with 1 µM Calcein AM and PI (as supplied) to stain live and dead cells, respectively. Fluorescence images were captured using the Celena S digital imaging system (Logos Biosystems, Villeneuve d’Ascq, France).

MTT Assay. Cell metabolic activity was determined using the MTT assay. Following 1 and 3 days of exposure to Pine bark extract solutions, 10 µL of MTT solution (5 mg/mL) was added to each well. Following 3 h of incubation at 37 °C, the medium was removed, and MTT-reduced formazan crystals were dissolved in DMSO for 15 min. Absorbance was measured at 550 nm using a Synergy HT microplate reader (Biotek). The viability of the control cells was set at 100%, and the relative viability of the treated groups was calculated accordingly.

### 2.5. Data Analysis

Statistical analysis concerning the characterization of Pine bark and Pine bark extracts was performed using Statistica for Windows software package, version 14.0.0.15 (TIBCO Software, Palo Alto, CA, USA). All analytical parameters were determined in triplicate. Differences between groups were determined using a T-test (independent, by groups). Data from biological studies are presented as mean ± standard deviation of three independent experiments, each one performed in triplicate. Differences between treatment groups were analysed using one-way ANOVA followed by Tukey’s post hoc test for multiple comparisons (SPSS software, version 28.0, IBM^®^). A *p*-value < 0.05 was considered statistically significant.

## 3. Results and Discussion

### 3.1. Pine Bark (PB) Characterization

#### 3.1.1. Particle Size Distribution

**Figure 1:**
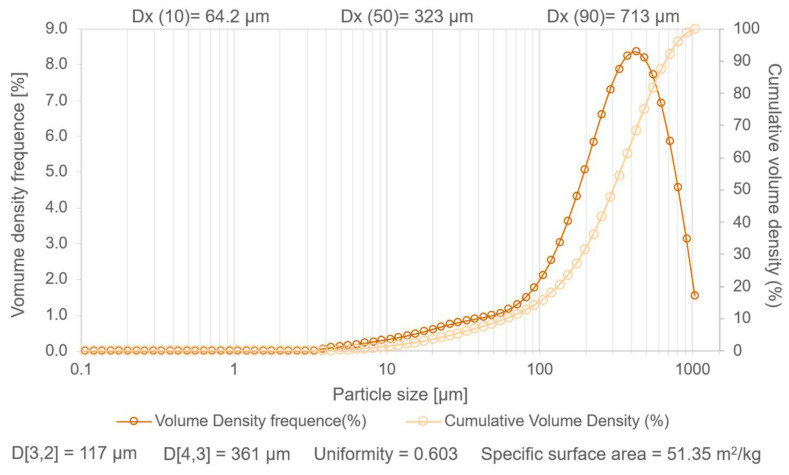
Particle size measurement of *Pinus pinaster* bark by laser diffraction granulometry.

The granulometry of the milled pine bark selected for this study was the fraction of particles with diameters ranging from 200 to 850 μm, obtained by the sieve procedure. In order to know the particle size more accurately, the size distribution was determined by laser diffraction granulometry (Figure 1).

The frequency curves of the volume and accumulated volume showed that there was a relatively large granulometric interval of roughly 64 µm (Dx (10)) to 713 µm (Dx (90)), i.e., indicating a considerable dispersion of particle size, which was supported by the uniformity value of 0.603. On the other hand, the Dx (50) value of 323 µm showed that half of the pine bark sample, in terms of volume, was below this order of magnitude. The large amplitude between D[4,3] (361 µm) and D[3,2] (117 µm)—meaning diameters of the particle size based on volume-weighted and area-weighted mean results, respectively—indicated the coexistence of very small particles and considerably larger ones. The measured surface area of 51.35 m^2^/kg (0.051 m^2^/g) showed a reasonable degree of grindability. Overall, it could be noted that the accumulated volume showed almost no particles smaller than 10 µm, with the highest frequency density values being above 100–200 µm, which is consistent with the values given by (Dx (50) and D[4,3]) of 323 µm and 361 µm, respectively. The value of Dx (10) is relatively distant from Dx (90), also corroborating that despite the existence of fine particles, with sizes in the order of 10 µm, there was a considerable volumetric fraction of larger particles, with dimensions close to 700 µm. Thus, the ground pine bark exhibited a broad particle size distribution, ranging from about 64 µm to 700 µm (main range), with a median size of roughly 300 µm and a specific surface area of approximately 50 m^2^/kg.

#### 3.1.2. Chemical Characterization

Table 2 displays the chemical composition of the pine bark (PB) employed in this study. The results characterize the PB that is being studied, as PB is influenced by a few factors, including the tree’s age, location, and growth conditions [37].

The ash content in PB was 0.44%, in agreement with values from other studies, notably by Alonso-Esteban et al. (2022) [8], who reported content below 1%, and Santos et al. (2021) [38] with 0.40%. The lipid and protein fractions showed values of 0.98% and 1.94%, respectively, with the fat value in the same order of magnitude as that presented by Santos et al. (2021) [38].

Softwood barks, such as maritime pine bark, were found to have a higher lignin content than most hardwood barks and lower acid-soluble lignin content [39,40]. The main component of the overall composition of PB was lignin, with 51.15% (48.31% Klason lignin and 2.85% acid-soluble lignin), values like those reported by Santos et al. (2021) [38] (46% for Klason lignin). The holocellulose content (46.09%) was comparable to the value reported by Fradinho et al. (2002) [41] (48.4%). Regarding cellulose and hemicellulose, the concentrations obtained were higher than those found by Santos et al. (2021) [38], although the α-cellulose content of 22.88% was similar to the 23.2% reported by the same authors (2021) [38].

The total extractives of the sample, 9.75% (*w*/*w*), showed the same order of magnitude as that reported by Nunes et al. (1996) [42] and Santos et al. (2021) [38], with 11.4% and 11.97%, respectively. The highest yield of extractives was obtained in the toluene–ethanol mixture (T:E) (5.61%), with the combination of toluene–ethanol and ethanol extractives presenting a value lower (7.15%) than the values in the literature, with the closest value (8.8%) reported by Nunes et al. (1996) [42]. This extractive fraction’s percentage in the overall extractives, notwithstanding the discrepancies, was 73.3%, falling within the range of values from other studies (77.2% [42] to 8.5% [38]). A lower hydrophilic extractive amount compared to the other extractives was indicated by a value of 2.59%, in line with the study by Nunes et al. (1996) [42]. In the present study, a sequential extraction procedure was applied using solvents of varying polarity: toluene–ethanol to extract nonpolar groups, water to remove polar structures, and ethanol for groups with similar solubility, as previously recommended [43].

The WHO recommends evaluating the levels of heavy metals in plants used as raw materials for finished products. Since pine bark extract can be incorporated into nutraceutical and cosmetic products, the concentrations of certain metals were analyzed (Table 2).

The elements Ca, Mg, K, Na, S, Fe, P, and Mn had the highest concentration in the inorganic fraction of pine bark (ash), with concentrations of each element above 34 mg/kg of dry matter (3.4 × 10^−3^% dry matter). Fe and Mn are essential elements as they affect the oxidation-reduction, photosynthesis, and nitrogen and nucleic acid metabolic processes. Excessive Mn hinders the mobility and absorption of Fe in plant tissues, and the opposite is also true—a high concentration of Fe decreases the effectiveness and absorption of other metals [44,45]. Sulfur is an important element for both protein structure [46] and enzyme function [47] and plays roles in maintaining the homeostasis of essential micronutrients such as Fe, Cu, Zn, and Mn [48] and in plant defense against stress and pests [47]. The elements Cu, Zn, Ni, and Cr are classified as micronutrients and play a crucial role as constituents of numerous plant enzymes [49] and plant growth regulators [45]. Their concentrations ranged from 0.33 to 3.11 mg/kg of dry matter (3.3 × 10^−5^ to 3.11 × 10^−4^% dry matter), values generally lower than those reported in other studies, as by Kirchner et al. (2008) [50] and Poikolainen (1997) [51], which seemed to indicate that the pine bark selected for this study was not greatly contaminated by them.

### 3.2. Chemical Characterization of Pine Bark Extract (PBE)

#### 3.2.1. Extraction Yield

Extractions performed with the two solvents (water and 1% DMSO) resulted in significantly different extraction yields (*p* < 0.01). Yield values were 5.77% and 6.55% (PBE dry matter/100 g PB), corresponding to 2.91 and 3.31 mg/mL (mg PBE/mL PBE), for water and 1% DMSO, respectively. The use of 1% DMSO produced an extract with greater dry matter quantity (a more concentrated extract), showing an increase of 13.7%, compared with that achieved with water.

The aqueous yield values (5.77%) exceeded those previously reported for extractions using water (2.4–3.2% *w/w* dry *P. pinaster* Ait. bark) [41,42,52]. In addition to the extraction medium, factors such as geographical origin, edaphoclimatic conditions, pine tree age, as well as sample preservation and extraction methods, can significantly influence extraction yields. For example, Pals et al. (2022) [53] obtained higher yields of extractives from pine bark (*Pinus sylvestris* L.) using water at 70–110 °C (with yields ranging from 12.6–19.9% for MAE) when compared to this study. In contrast, Galili and Hovav (2014) [54] reported that temperatures above 100 °C can cause a significant reduction in the extraction yield but improve the extraction efficiency of phenolic compounds by softening plant tissues, weakening the cell wall, increasing the solubility of polyphenols, and increasing the diffusion and mass transfer rates of the extracted compound. Moreover, elevated temperatures reduce the viscosity of the solvent, increasing its penetration into the sample matrices, improving the extraction rate.

#### 3.2.2. FTIR Spectroscopy

**Figure 2:**
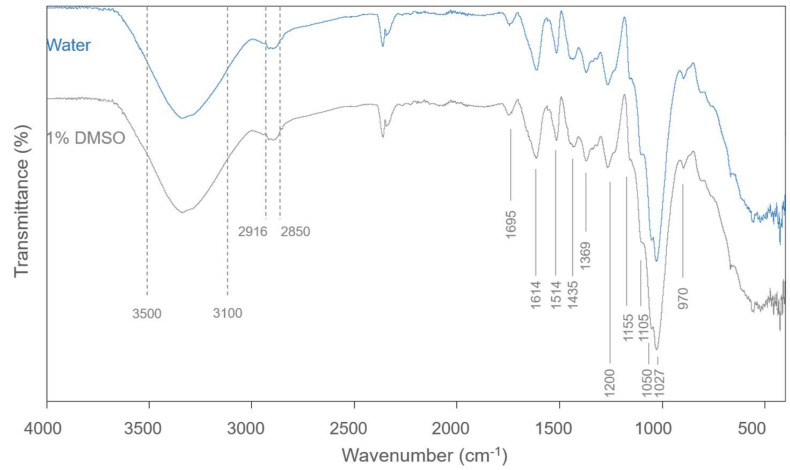
FTIR spectra of 100% Water and 1% DMSO extracts from *Pinus pinaster* bark.

The FTIR spectra, in the wavenumber range of 500 to 4000 cm^−1^, of the extracts obtained by the two solvents (water and 1% DMSO) are shown in Figure 2. This technique was used to investigate the presence of polyphenols in pine bark extract samples. The infrared spectra of the extracts displayed similar profiles, with no notable variations between them.

There are some characteristic bands that can be related to specific organic functional groups. The band in the range 3500–3100 cm^−1^ is related to the –OH stretch vibration in phenolic and aliphatic structures. Bands associated with the –CH stretch vibration in aromatic methoxy groups and in side-chain methyl and methylene groups were displayed in the spectra at 2916 and 2850 cm^−1^. The band exhibited at 1695 cm^−1^ can be attributed to the conjugated carbonyl-carbonyl stretching. The bands displayed at 1614, 1514, and 1435 cm^−1^ matched to aromatic skeleton vibrations and to –CH deformation (1435 cm^−1^). Furthermore, the 1514 cm^−1^ band also points to the existence of non-gallate procyanidins. The band located at 1369 cm^−1^ is assigned to the phenolic stretch vibration of OH and the deformation of aliphatic CH in methyl groups. The peaks displayed at 1200 cm^−1^ and 1050 cm^−1^ are attributed to asymmetric and symmetric stretching vibrations of C–O, and the ones displayed at 1155 and 1105 cm^−1^ to aromatic –CH in-plane bending vibrations, indicating aromatic ring deformations and interactions with substituents of rings, due to structures of phenols and flavonoids. The band located at 1027 cm^−1^ can be ascribed to aliphatic C–O stretching. The band at 970 cm^−1^ is associated with an aliphatic C–OH stretching vibration. Aromatic–CH stretch vibrations are exhibited in bands at wavelengths lower than 900 cm^−1^. Bands in the range 860 to 775 cm^−1^ are assigned to the stretching and bending vibrations of –CH from aromatic rings, being associated with phenolic compounds, which is corroborated by the bands exhibited in the range 1600–1500 cm^−1^. The group of identified peaks suggests the presence of phenolic compounds in the samples analyzed. The interpretation of IR spectra was guided by the studies of Chupin et al. (2013, 2015) [55,56] and Santos et al. (2021) [38].

#### 3.2.3. Content of Total Polyphenol and Total Flavonoid

Plants synthesize a diverse array of phenolic antioxidants, including flavonoids, proanthocyanidins, cinnamic acids, benzoic acids, coumarins, stilbenes, lignans, and lignins. The antioxidant properties of polyphenols are primarily determined by their chemical structure, hydrogen bonding potential, metal ion chelation and reduction abilities, solvent interactions, adduct formation, and reduction potential [57].

Water extraction enables the concentration of hydrophilic aromatic compounds. Further, microwave-assisted water extraction (MAE) is highly efficient in isolating aromatic compounds, achieving results comparable to those obtained with organic solvents [53]. Korkalo et al. (2020) results indicate that deionized water is the most suitable solvent for microwave-assisted extraction of tree bark, due to its high microwave absorbance capacity, strong ability to dissolve phenolic metabolites, and lack of environmental harm [58].

Total phenolic content (TPC) and total flavonoid content (TFC) are two methods used to evaluate the potential antioxidant capacity of plant materials, or the antioxidant activity of their derived extracts [59].

**Figure 3:**
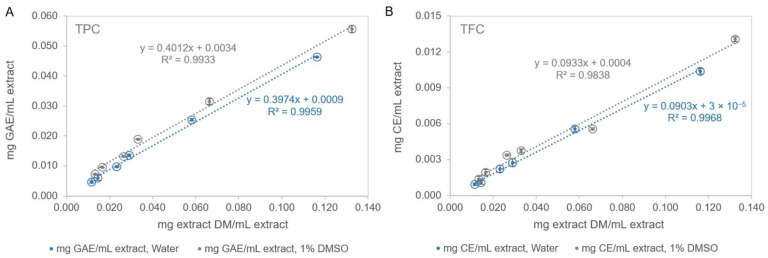
(**A**)—Total phenolic content (TPC) and (**B**)—Total flavonoid content (TFC) of the 100% water and 1% DMSO aqueous extracts of *Pinus pinaster* bark. Values shown: mean ± SD of 3–4 replicates; GAE—gallic acid equivalents; CE—catechin equivalents.

The TPC content of the aqueous extracts, i.e., 100% Water and 1% DMSO of pine bark, is shown in Figure 3A. The results showed high and comparable values for both extracts (397.4 ± 5.7 and 401.2 ± 8.1 mg GAE/g PBE, for the Water and 1% DMSO extracts, respectively; *p* > 0.05). Polyphenols are key components of pine bark extracts, as they are directly linked to the overall antioxidant activity [14].

When expressed as mg GAE per g of extract, Ramos et al. (2022) [9] reported a TPC of 539 mg GAE/g extract from *P. pinaster* Ait. extracts. In comparison, Chupin et al. (2015) [56] described values ranging from 236 to 306 mg GAE/g extract, depending on the extraction conditions, while Dudonné et al. (2009) [60] and Ustun et al. (2012) [61] reported values of 363 mg GAE/g in a commercial extract and 572.28 mg GAE/g extract for Pycnogenol^®^, respectively. Additionally, the TPC values reported for hot water extracts (HWEs) of barks from other *Pinus* species range from 111 mg GAE/g extract for *P. rigida* × *taeda* to 862 mg GAE/g extract for *P. koraiensis* [14].

For TPC expressed as mg GAE/g *P. pinaster* bark, this study reported 22.93 and 26.27, respectively, in water and 1% DMSO. Comparatively, Chupin et al. (2015) [56] described values of 8 to 39 mg GAE/g *P. pinaster* bark, depending on extraction conditions, with the highest yield obtained for a particle size of 50–100 µm and 80% ethanol as solvent. In a separate study, Chupin et al. (2013) [55] recorded TPC values between 22 and 62 mg GAE/g bark. Ramos et al. (2022) [9] found a TPC of 116 mg GAE/g *P. pinaster* bark, while Vieito et al. (2019) [62] reported a phenolic content of 50.09 mg GAE/g bark using Soxhlet extraction with water as the solvent. Using the same method of extraction (i.e., MAE) and 96.6% ethanol, but bark of another species (*P. abies*), Sládková et al. (2016) [24] reported 3.211 mg GAE/g bark as the highest value of TPC, lower than that obtained in the present study.

Despite variations in extraction conditions, aqueous extracts contain a substantial number of phenolic compounds with high bioactive or functional potential. Given its environmentally friendly nature, water should be considered a promising solvent for phenolic extraction.

The concentration of total flavonoids (TFC) was much lower than that obtained in TPC, i.e., only about 22% of TPC were flavonoid compounds, with these parameters being related by the equation, mg GAE/mL PBE=4.3051×mg CE/mL PBE+0.0015 (R^2^ = 0.9822; *p* < 0.001). The values found for TFC were similar in both extracts (*p* > 0.05) with values of 90.3 ± 2.0 and 93.3 ± 2.3 mg CE/g PBE, for the solvent 100% Water and 1% DMSO, respectively (Figure 3B). The aqueous extract of the bark of the *Larix decidua* tree showed a TFC value of 34 ± 2 mg CE/g extract in the study by Sillero et al. (2021) [63], while Hamad et al. (2019) [64] reported values of 22 mg CE/g extract, obtained from a 65:35 methanol-water mixture (*v*/*v*), for *Pinus sylvestris* bark and 33.4 mg CE/g PBE for *Pinus roxburghii* bark, values lower than those achieved in this study. In Ustun et al. (2012) [61] work, extracts obtained from various pine species (*P. brutia*, *P. pinea*, *P. nigra*, *P. halepensis*, and *P. sylvestris*) under different extraction conditions exhibited either no flavonoids or only low total flavonoid content.

A crucial property for chemical compound extraction is the polarity of both the chemicals to be extracted and the extraction solvent. A wide range of compounds with different chemical properties, including polarity, is found in the polyphenol group. Thus, the effectiveness of its extraction depends on factors such as the solvent/sample ratio, sample type, extraction method, extraction temperature and time, and extraction solvent. In terms of solvents, extracts produced by solvents with different polarities have differences in amounts and compositions [65]. More polar solvents should extract more polar molecules [65], with the affinity for the solvent determining the compound’s solubility and potentially limiting the maximum extractable quantity [66]. A reason for the discrepancy between TPC and TFC concentrations could therefore be the low water solubility of flavonoid molecules as a result of their structure. These compounds have a strong nonpolar character due to their large and complex structure with a high number of carbon atoms, even if they may have oxygen atoms in the side chains for hydrogen bonding with water [67]. Conversely, the solubility in water is higher for smaller phenolic compounds like ferulic acid, caffeic acid, and 3,4-dihydroxybenzoic acid [65].

#### 3.2.4. Antioxidant Activity

Antioxidants can be classified as “primary antioxidants” or “secondary antioxidants”. The first mechanism actively inhibits oxidation reactions, while the second acts indirectly. Phenolic compounds, as primary antioxidants, operate through two main reaction pathways: single-electron transfer (SET) and hydrogen-atom transfer (HAT). In the SET mechanism, an antioxidant donates a single electron to reduce target molecules, whereas in the HAT mechanism, the antioxidant scavenges free radicals by donating hydrogen atoms. Notably, SET and HAT reactions can occur simultaneously. In this study, antioxidant activity and the underlying mechanisms were evaluated using multiple assays: ORAC, based on the HAT mechanism and reflecting radical chain-breaking capacity; FRAP, based on SET through the reduction of the Fe^3+^ complex to Fe^2+^; and DPPH and ABTS, which operate through mixed HAT/SET mechanisms to measure free radical scavenging capacity [59,68].

While the ABTS and DPPH tests can measure both hydrophilic and lipophilic antioxidants, others only measure hydrophilic antioxidants (FRAP) [68]. All methods used are in vitro assays that provide overall or integrated parameters rather than quantifying individual antioxidants. Hydrophilic and lipophilic assays conducted in solvents of varying polarity allow for the comparison of antioxidant properties among different compounds (e.g., antioxidative vitamins, polyphenols, and secondary plant metabolites) and provide an initial assessment of the antioxidant capacity of food extracts and physiological materials. It is strongly recommended to employ at least two different methods, preferably with distinct mechanisms, and to use three sample dilutions within each assay [69].

**Figure 4:**
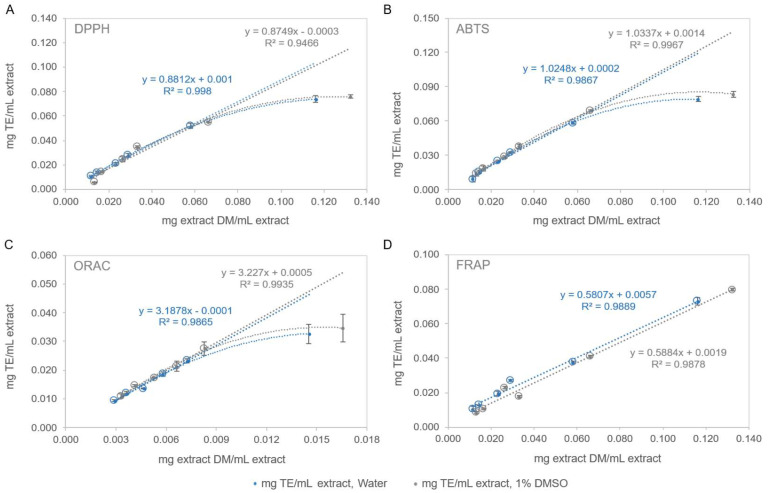
Antioxidant activity ((**A**)—DPPH, (**B**)—ABTS, (**C**)—ORAC, and (**D**)—FRAP tests) of the 100% water and 1% DMSO aqueous extracts of *Pinus pinaster* bark. Values shown: mean ± SD of 3 replicates; TE—trolox equivalents.

In this work, the antioxidant activity of the two pine bark extracts (100% water and 1% DMSO) was evaluated with different assays across a range of dilutions, as presented in Figure 4.

For both extracts analyzed, the results indicated comparable antioxidant activity across all tests performed, i.e., DPPH, ABTS, ORAC, and FRAP. The highest antioxidant activity was observed in the ORAC test (HAT mechanism), with values of 3188 ± 137 and 3227 ± 301 mg TE/g PBE for the 100% water and 1% DMSO extracts, respectively (Figure 4C). In contrast, the lowest activity was recorded in the FRAP assay (SET mechanism) with values of 581 ± 16 and 588 ± 11 mg TE/g PBE for the same extracts (Figure 4D).

In the DPPH (Figure 4A) and ABTS (Figure 4B) assays (mixed HAT/SET mechanism), both extracts exhibited higher values in the ABTS test. Specifically, the ABTS results were 1025 ± 41 and 1034 ± 17 mg TE/g PBE, compared to 881 ± 55 and 875 ± 13 mg TE/g PBE for the DPPH test in the 100% water and 1% DMSO extracts, respectively. Thus, ABTS demonstrated stronger scavenging activity than DPPH, with IC_50_ values of 49.31 and 47.66 µg/mL for ABTS versus 56.95 and 58.61 µg/mL for DPPH, in the 100% water and 1% DMSO extracts, respectively (with Trolox (TE) as a reference compound for both tests).

Aqueous extracts of *Pinus* maritima bark analyzed by Dudonné et al. (2009) [60] presented lower radical scavenging capacities in the DPPH and ABTS tests (IC_50_ = 94.51  ±  0.01 and IC_50_ = 76.71  ±  0.37 µg/mL, respectively) and also lower ORAC (6506  ±  120 µmol/g → 1628 ± 30 mg/g extract) compared to the values obtained in this study. The extract reducing power value in the same study was higher (6.45 mmol Fe^2+^/g extract → 377 mg Fe^2+^/g extract) than those found in the present study for both extracts (265 ± 12 and 265.8 ± 2.0 mg Fe^2+^/g extract for 100% water and 1% DMSO extracts, respectively).

Ramos et al. (2022) [9], in their study on polar extracts (extraction solvent: methanol/water/acetic acid (49.5:49.5:1, *v*/*v*/*v*)) of *P. pinaster* Ait. bark, reported the scavenging effects of DPPH (IC_50_ = 6.79 µg/mL) and ABTS (IC_50_ = 3.95 µg/mL) to be greater compared to those observed in the aqueous extracts from this study. This is in line with the comparative study of the activity of hydroethanolic and aqueous extracts by Venkatesan et al. (2019) [70], where they reported that ethanolic and hydroethanolic extracts had higher scavenging capacities (DPPH and ABTS) and higher values in the FRAP test compared to aqueous extracts. However, it should be noted that microwave-assisted extraction with water appeared to have a promising effect on the elimination of DPPH and ABTS free radicals. This outcome may be attributed to the different properties of the two radicals. Owing to its cationic nature, the ABTS radical can function as both an electron acceptor and a proton acceptor, whereas DPPH is a nitrogen-centered radical characterized by an unpaired electron. Another important distinction is that ABTS is less selective, reacting with a wide range of hydrogen donors, while DPPH interacts specifically with flavonoids that contain hydroxyl groups on both the aromatic ring and the B ring [71].

The antioxidant activity assays—DPPH, ABTS, ORAC, and FRAP—conducted on the two extracts revealed statistically significant differences (*p* < 0.05) across all tests (Table 3). The findings suggest that no single assay can fully capture the overall antioxidant activity of a sample, as each test has distinct mechanisms and inherent limitations. Thus, employing multiple antioxidant assays offers a more comprehensive and reliable evaluation, allowing for a more robust characterization of the sample’s potential to counteract oxidative processes.

The ranking of antioxidant activity differed according to the assay applied. Because the methods used to determine total phenolic and flavonoid content, as well as antioxidant activity, are strongly influenced by reaction conditions and by the chemical nature of the substrates and products, the activity values obtained may vary considerably between assays [4,69].

**Figure 5:**
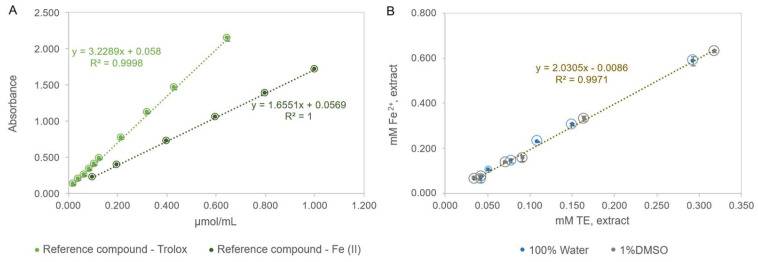
FRAP assay: (**A**)—Calibration curves with Trolox and Fe (II) as reference compounds; (**B**)—Relationship between the antioxidant activity values obtained for the two extracts by the FRAP assay, using Trolox and Iron (II) sulphate as reference compounds in the calibration curve.

In the FRAP assay, the results are typically expressed either as µmol/mL of TE or as µmol/mL of Fe^2+^. Consequently, when interpreting and comparing the antioxidant activity data across studies, it is essential to account for differences in the reporting units. In calibration curves constructed with two reference compounds, Trolox and Fe^2+^ (FeSO_4_), the absorbance observed for a given Fe^2+^ concentration was approximately half that of the equivalent molar concentration of Trolox $(Absorbance=1.6551\times µmol\;{Fe}^{2+}/mL+0.0569,\;R^{2}=1;\;Absorbance=3.2289\times µmol\;Trolox/mL+0.058,\;R^{2}=0.9998$) (Figure 5A). The antioxidant activity of the two extracts (100% water and 1% DMSO), evaluated by the FRAP assay using calibration curves with Trolox and iron (II) sulfate as reference compounds, indicated that the relative antioxidant activity of Trolox was approximately twice that of Fe^2+^ (Figure 5B), consistent with the findings of Galili and Hovav (2014) [54]. The data obtained for the reference compounds Trolox and Fe^2+^ can be interconverted using the following mathematical equation, $mM\;{Fe}^{2+}=2.0305\times mM\;Trolox-0.0086$ (R^2^ = 0.9971).

#### 3.2.5. Assessment of Extract Similarities

The two extracts showed no significant differences (*p* > 0.05) in any of the evaluated parameters when expressed as mg/g PBE (*w*/*w*). However, when the results were expressed as mg/mL PBE (*w*/*v*) or mg/g PB (*w*/*w*), statistically significant differences (*p* < 0.05) were observed between the extracts for all parameters (Table 4).

The differences observed in the extract concentrations across parameters were attributable to the significantly different extraction yields of the two extracts (*p* < 0.05). When expressed as mg/mL of extract, the concentrations of TPC, TFC, and antioxidants were consistently higher (*p* < 0.05) in the 1% DMSO extract, owing to its greater concentration (3.10 ± 0.01 mg/mL) compared with the 100% aqueous extract (2.91 ± 0.02 mg/mL). A similar pattern was evident when results were expressed as mg/g of dry pine bark, since producing the same extract mass required less pine bark for the 1% DMSO extract than for the aqueous extract.

Correlations were calculated for both extracts across the various parameters, except for ORAC. Correlations between ORAC results and the other tests could not be performed because the ORAC assay required higher sample dilutions than those used for the other parameters. Statistically significant correlations were observed between the results for both extracts in all assays. The evaluated parameters exhibited high coefficients of determination (R^2^), ranging from 0.8845 to 0.9971. However, the values of the measured parameters differed significantly from one another (*p* < 0.05) (Table 5).

The lowest R^2^ values were observed between the DPPH (mixed HAT/SET mechanism) and FRAP (SET mechanism) assays, with values of 0.8923 and 0.8845 for Trolox and Fe^2+^ as reference compounds, respectively. Overall, a strong correlation was found between the total phenolic and flavonoid contents of the bark extracts and antioxidant activity measured by DPPH, ABTS, and FRAP (R^2^ = 0.9472 for TFC vs. DPPH to 0.9832 for TPC vs. ABTS, minimum and maximum values, respectively), suggesting that these extracted bioactive compounds significantly contribute to the observed antioxidant effects. This contrasts with the findings of Ku (2007), who reported low correlations attributed to the influence of individual antioxidant components and the presence of polar reducing or non-phenolic oxidizable impurities, such as acids and sugars [14].

#### 3.2.6. Phenolic Compound Profile by HPLC-DAD Analysis

A total of 10 phenolic compounds were identified in the extracts based on solutions of the corresponding standards (Table 6). Taxifolin and catechin were the phenolic compounds that showed the highest concentrations in both extracts, with respective concentrations of 54.1 ± 2.7 mg/L and 62.1 ± 4.3 mg/L for the water extract and 63.9 ± 2.0 mg/L and 72.1 ± 1.6 mg/L for the 1% DMSO extract (each compound contributed approximately 30% to the quantified phenolic content). The same pattern of behaviour was observed in the bark extracts of *P. densiflora* by Kim et al. (2025) [72] and *P. pinea*, *P. sylvestris*, and *P. pinaster* by Karaçelik et al. (2022) [73]. In pine bark extracts of *P. pinea*, *P. sylvestris*, *P. nigra*, *P. parviflora*, and *P. ponderosa*, Yesil-Celiktas et al. (2009) [74] also detected these chemicals in high amounts and suggested that this pattern might be connected to climatic stress conditions. The concentrations of the other phenolic compounds ranged from 3.64 ± 0.25 mg/L to 17.22 ± 0.52 mg/L and from 4.370 ± 0.096 mg/L to 19.8 ± 1.1 mg/L (caffeic acid and protocatechuic acid concentrations) for the aqueous and 1% DMSO extracts, respectively. The identified and quantified phenolic compounds in the PBE samples were in agreement with the results for water extracts in the literature (Alonso-Esteban et al., 2022) [8]. Since water was used as the extraction solvent in this study, phenolic compounds that are poorly soluble in water would have been extracted in very small amounts [65,66], which is why they were either not detected at all or just weakly detected. Rich in phenolic compounds, including flavonoids, these extracts, obtained using non-toxic solvents, can be used as added-value products in the food and nutraceutical sectors.

The individual phenolic compounds of both extracts displayed patterns similar to the aggregated constituents (TPC, TFC) and the antioxidant activity observed across the different assays. Statistically significant differences (*p* < 0.05) were observed when the results were expressed as mg/mL of PBE (*w*/*v*) or mg/g of PB (*w*/*w*), whereas no significant differences (*p* > 0.05) were found when reported as mg/g of PBE (*w*/*w*).

### 3.3. Antibacterial Activity and Cytotoxicity of the Aqueous Extract

As shown above, the 100% aqueous and 1% DMSO pine bark extracts exhibited comparable total phenolic and flavonoid contents, as well as similar antioxidant activities when expressed per gram of PBE (*w*/*w*). The results demonstrated that the 100% aqueous extract is endowed with antioxidant activity, as demonstrated by its TPC and TFC and by chemical assays including DPPH, ABTS, and FRAP (evaluated at 0.0116 to 0.116 mg/mL extract), as well as ORAC (0.0029 to 0.0145 mg/mL extract). Phenolic compounds were also identified in the extract. Based on these findings, the 100% water extract was assessed for the antibacterial activity and cytotoxicity toward eukaryotic cells over a broad concentration range (0.0029–1.4500 mg/mL, corresponding to dilutions of 1/1000 to 1/2 of the initial extract).

#### 3.3.1. Antibacterial Activity

**Figure 6:**
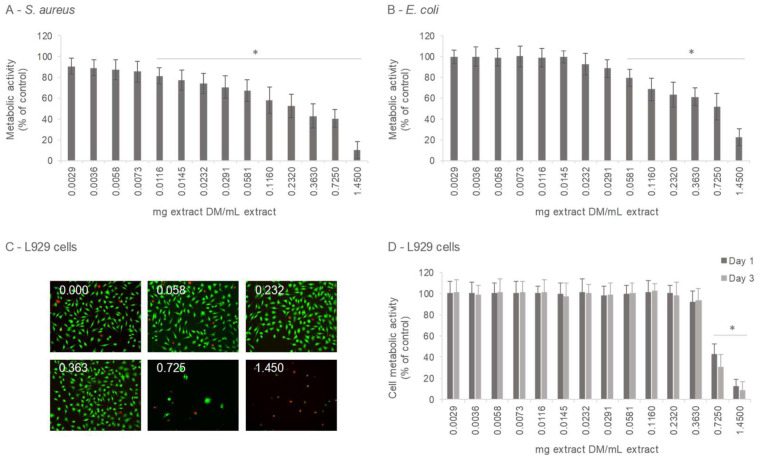
Antibacterial activity and cytotoxicity of *Pine pinaster* aqueous extract (100% Water; 0.00 to 1.450 mg extract/mL extract): Activity against *S. aureus* (**A**) and *E. coli* (**B**), 1 day exposure; cytotoxicity toward L929 fibroblast cells: (**C**)—live/dead assay (0.00, 0.058, 0.232, 0.363, 0.725 and 1.450 mg extract/mL extract), 1 day exposure); (**D**)—metabolic activity, 1 and 3 days exposure. * Significantly different from control (absence of extract): *p* < 0.05.

Antibacterial activity of the 100% water extract, tested across a dilution range, was assessed against the Gram-positive *S. aureus* and the Gram-negative *E. coli*, with the results shown in Figure 6A,B. Compared to control (absence of extract), significant concentration-dependent inhibition was achieved at levels ≥ 0.0116 mg/mL extract (1/250 dilution) against *S. aureus* and ≥0.0581 mg/mL extract (1/50 dilution) against *E. coli*, indicating greater susceptibility of *S. aureus*. These results are consistent with previous reports showing that Gram-positive bacteria are generally more susceptible to inactivation by pine extracts than Gram-negative species [75,76]. In the case of *P. pinaster* Ait., several studies proved the antibacterial activity of its extracts against both tested microorganisms. Polar extracts have been shown to inhibit *S. aureus* and *E. coli*, with stronger effects observed against the Gram-positive strain [9]. Conversely, aqueous PB extracts were reported to inhibit *S. aureus* without affecting *E. coli* [77]. In addition, Pycnogenol^®^ has demonstrated suppressing activity against methicillin-resistant *S. aureus* [78], while earlier work by Torras et al. revealed bacteriostatic effects on both *S. aureus* and *E. coli* [79].

#### 3.3.2. Cytotoxicity/Cytocompatibility

The cytotoxicity of the 100% aqueous extract was assessed in L929 mouse fibroblast cultures exposed to a concentration range of the extract for 1 and 3 days. L929 cells are commonly used in cytotoxicity testing of plant extracts, following ISO Guidelines (ISO 10993-5:2009; [80]), which consider a reduction of more than 30% in cell viability (relative to untreated controls) as cytotoxic. In this study, no cytotoxic effects were observed at concentrations up to 0.363 mg/mL (1/8 dilution of the original extract). A concentration-dependent decline in viability emerged only at ≥0.725 mg/mL (1/4 dilution), as determined by the live/dead assay at day 1 (Figure 6C) and the MTT assay at days 1 and 3 (Figure 6D). Overall, these findings demonstrate that the aqueous extract exhibits high cytocompatibility with eukaryotic cells. The use of L929 cells is advised for an initial cytotoxicity screening as stated by the ISO 10993-5 guidelines. However, data from an additional relevant cell type (e.g., normal human epithelial or fibroblast cells) will provide stronger evidence of the cytocompatibility of the Pine bark extracts. In vitro investigations on the cytotoxicity of *P. pinaster* Ait. extracts in normal cells remain scarce, as most studies have concentrated on their effects in cancer cell lines [81]. Evidence suggests that pine bark extract may exert selective cytotoxicity toward certain cancer cells, consistent with broader findings that natural extracts can inhibit abnormal cell proliferation while sparing normal cells. The low toxicity profile of pine bark extracts is further supported by their established clinical use, reinforcing their potential health-promoting benefits [11,82].

**Figure 7:**
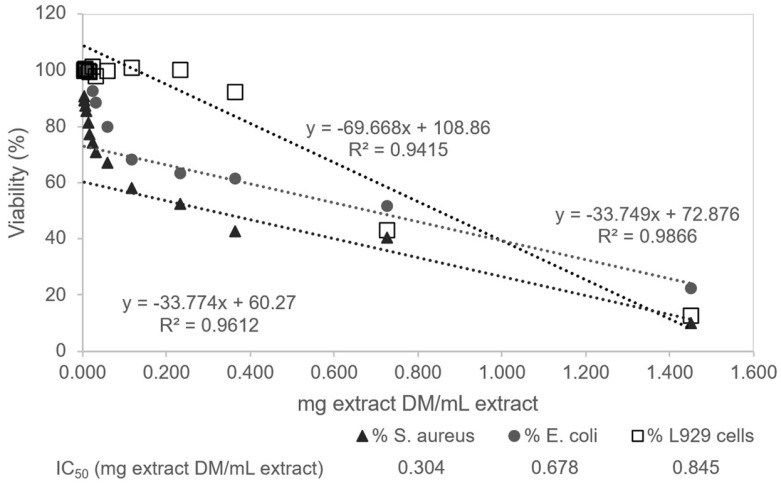
Comparison of the inhibitory effects of *Pinus pinaster* 100% water extract on the viability of fibroblast cells, *S. aureus*, and *E. coli*.

The results presented in Figure 6 show that the 100% water extract exhibited antibacterial activity in a concentration range that is not cytotoxic to eukaryotic cells. Comparison of the inhibitory effects of the extract regarding the viability of fibroblast cells, *S. aureus*, and *E. coli* is presented in Figure 7. Cytocompatibility (absence of toxicity) to fibroblast cells was observed in the range 0.0029 to 0.363 mg/mL extract. Within this cytocompatible range, antibacterial activity was observed at concentrations ≥ 0.0116 mg/mL for *S. aureus* and ≥0.0581 mg/mL for *E. coli*, indicating selective antibacterial effects. The antibacterial IC_50_ values were 0.304 mg/mL for *S. aureus* and 0.678 mg/mL for *E. coli*, compared with the IC_50_ of 0.845 mg/mL for fibroblast cells, highlighting the higher susceptibility of prokaryotic cells relative to eukaryotic cells.

#### 3.3.3. Correlation Between Antioxidant Activity and Biological Effects

**Figure 8:**
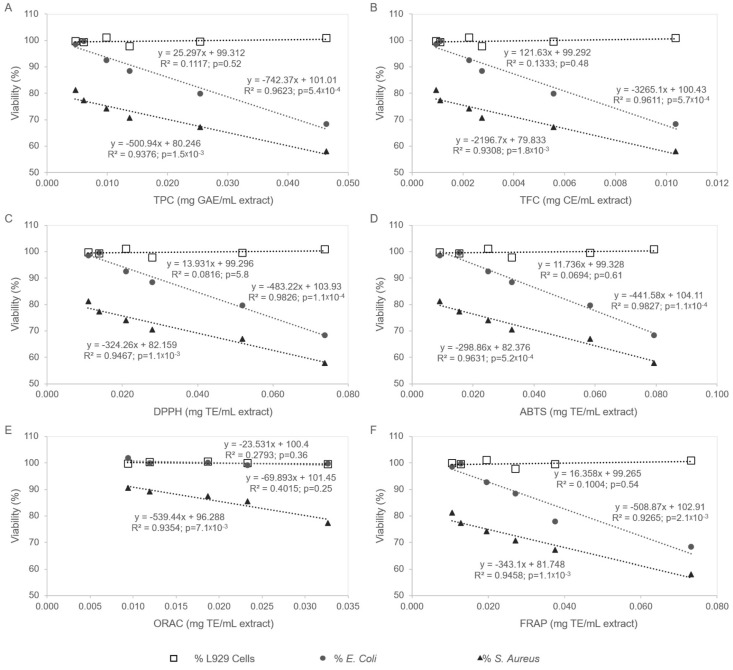
Correlation between antioxidant and antibacterial activities in *Pinus pinaster* aqueous extract (*p* < 0.05). No correlation was detected concerning antioxidant activity and fibroblast cytotoxicity (*p* > 0.05). (**A**) Total Phenolic Content, TPC; (**B**) Total Flavonoid Content, TFC; (**C**) DPPH; (**D**) ABTS; (**E**) ORAC; (**F**) FRAP.

Correlation between antioxidant activity and biological outcomes, namely cytocompatibility and antibacterial activity, is illustrated in Figure 8. Correlation analyses were conducted within the concentration range of 0.0116–0.116 mg/mL for total phenolic and flavonoid contents, as well as for the DPPH, ABTS, and FRAP assays, and within 0.0029–0.0145 mg/mL for the ORAC assay. The results revealed a positive association between antioxidant and antibacterial activities in a concentration range that remained non-toxic to fibroblast cells. With the exception of the ORAC assay, significant positive correlations (*p* < 0.05) were observed between antioxidant activity and the inhibitory effects against *S. aureus* and *E. coli*. For the ORAC assay, evaluated within a lower concentration range, a positive correlation was found only for *S. aureus* (*p* < 0.05). These findings are consistent with previous reports suggesting that phenolic compounds contribute to the antibacterial activity of pine bark polar extracts [83]. Polyphenols constitute a diverse class of secondary plant metabolites characterized by the presence of at least one phenolic ring bearing one or more hydroxyl groups. Depending on the specific compound, additional functional groups may also be present. Due to their structural diversity and complex antibacterial mechanisms, polyphenols can exhibit synergistic effects, particularly in extract preparations. Among their structural features, hydroxyl groups are considered key determinants of antimicrobial activity, as they enable hydrogen bonding with cellular components, primarily promoting accumulation on the bacterial surface [84]. The number and position of hydrophilic side chains appear to be proportional to the strength of polyphenol interactions with bacterial cells [85]. Based on their degree of hydrophobicity, polyphenols may accumulate on the cell wall, interact with the cytoplasmic membrane, and even penetrate into the cytoplasm [85]. These interactions can result in various cellular effects, including cell wall damage, loss of structural integrity, reduced mechanical strength, and decreased resistance to osmotic stress. Additionally, polyphenols can disrupt the structure and function of the cell membrane, which contains numerous enzymes and systems essential for physiological processes, thereby increasing membrane permeability and causing leakage of intracellular contents. Once inside the cytoplasm, polyphenols may interfere with several critical pathways, such as inhibiting DNA gyrase and disturbing DNA replication, RNA and protein synthesis, and intermediary metabolism involved in energy generation, mechanisms that have been identified for catechins [85,86]. For example, phenolic acids, detected in the tested aqueous extract, can cross the cytoplasmic membrane via passive diffusion of their undissociated forms, leading to localized membrane acidification, pore formation, cytoplasmic penetration, and intracellular acidification. Polyphenols may also act as pro-oxidants by stimulating bacterial reactive oxygen species (ROS) production, thereby inducing oxidative stress within the intracellular environment, a mechanism observed for catechins and proanthocyanidins, also present in the tested extract [85,86,87,88,89]. Ultimately, this oxidative damage can trigger apoptotic-like cell death as a generalized response to cellular injury [85]. In contrast, no correlation was detected between antioxidant activity and fibroblast cytotoxicity (*p* > 0.05), an interesting result indicating that the aqueous extract retains its antioxidant properties without compromising eukaryotic cell viability.

### 3.4. Limitations of This Study

Despite providing valuable insights, this study is limited by its in vitro experimental design, which may not fully replicate the complexity of in vivo biological systems. The antioxidant and antibacterial activities of PBE were assessed under controlled laboratory conditions that do not account for factors such as bioavailability, metabolism, or interactions within tissues and bodily fluids. The cytocompatibility assays were performed using a single eukaryotic cell type, which may not represent the full spectrum of cellular responses in different tissues. Additionally, the antibacterial evaluation was restricted to two bacterial strains, limiting the generalization of antimicrobial efficacy. The chemical characterization relied primarily on spectrophotometric and HPLC-DAD analyses, which may not capture the complete profile or potential synergistic interactions of bioactive compounds. Therefore, further studies, particularly in vivo investigations and broader antimicrobial screening, are required to confirm the biological relevance of aqueous PBE.

## 4. Conclusions

The aqueous bark extract of *Pinus pinaster* Ait., obtained via microwave-assisted extraction, demonstrated strong antioxidant activity, supported by its phenolic and flavonoid content and confirmed through four complementary assays (DPPH, ABTS, ORAC, and FRAP), which showed high inter-method correlations. The extract also exhibited antibacterial activity against *S. aureus* and *E. coli*, with Gram-positive bacteria being more susceptible. Notably, within the cytocompatible range to fibroblast cells, the extracts exhibited both antioxidant and antibacterial effects, underscoring their potential for applications involving direct interaction with eukaryotic cells. These results highlight water as a sustainable green solvent and suggest that *P. pinaster* aqueous extracts are a high-value product with promising uses in food and nutraceutical applications.

## Figures and Tables

**Table 1:** HPLC-DAD—standard compounds, determination coefficients (R^2^), and limits of detection (LoD) of phenolic compounds.

Compound	Hill Notation	Wavelength (nm) ^1^	R^2^	LoD (mg/L)
Cinnamic acid	C_9_H_8_O_2_	280	0.9996	0.079
Catechin	C_15_H_14_O_6_	280	0.9995	0.197
Gallocatechin	C_15_H_14_O_7_	280	0.9956	0.995
Epicatechin	C_15_H_14_O_6_	280	0.9997	0.671
Quercetin	C_15_H_10_O_7_	320	0.9993	0.070
Gallic acid	C_7_H_6_O_5_	280	0.9998	0.248
Syringic acid	C_9_H_10_O_5_	280	0.9997	0.342
Caffeic acid	C_9_H_8_O_4_	320	0.9992	0.143
Taxifolin	C_15_H_12_O_7_	280	0.9992	0.383
Ferulic acid	C_10_H_10_O_4_	320	0.9999	0.051
Ellagic acid	C_14_H_6_O_8_	280	0.9984	0.556
Protocatechuic acid	C_7_H_6_O_4_	280	0.9990	0.378
Vanillin	C_8_H_8_O_3_	280	0.9998	0.185
Resveratrol	C_14_H_12_O_3_	320	0.9999	0.059
o-coumaric acid	C_9_H_8_O_3_	280	0.9996	0.079

^1^ Wavelength used in HPLC-UV quantification.

**Table 2:** Chemical composition of dry pine bark from *P. pinaster* Aiton subsp. *atlantica*, expressed as a percentage of dry matter (DM) weight (% *w*/*w*, g/100 g).

Parameter	(% *w*/*w*)
Moisture	6.67 ± 0.01
Total protein	1.94 ± 0.22
Total fat	0.98 ± 0.00
Ash	0.44 ± 0.01
Holocellulose	46.09 ± 0.48
Cellulose	23.21 ± 0.57
α-Cellulose	22.88 ± 0.02
Hemicellulose ^a^	23.21 ± 0.02 ^a^
Lignins	51.15 ± 0.35
Acid soluble	2.85 ± 0.35
Klason	48.31 ± 0.31
Total extractives	9.75 ± 0.13
Toluene–ethanol (T:E)	5.61 ± 0.05
Ethanol (E)	1.54 ± 0.06
Water (W)	2.59 ± 0.10
Minerals	
Minerals, Content > 3.4 × 10^−3^% ^b^	1.52 × 10^−1^ ± 1.91 × 10^−3^
Minerals, Content < 3.2 × 10^−4^% ^c^	5.01 × 10^−4^ ± 1.12 × 10^−5^

^a^ Hemicellulose = (holocellulose − α-cellulose); ^b^ ∑ mineral concentration; minerals: Ca, Mg, K, Na, S, Fe, P, Mn; (1523 ± 19 mg/kg dry matter); ^c^ ∑ mineral concentration; minerals: Zn, Cu, Cr, Ni; (5.01 ± 0.11 mg/kg dry matter).

**Table 3:** T-test (*p* values) for independent samples.

		DPPH	ABTS	ORAC	FRAP
mg TE/g PBE	DPPH	-	2.17 × 10^−2^	1.10 × 10^−5^	7.95 × 10^−4^
(PBE—100% Water)	ABTS	2.17 × 10^−2^	-	1.30 × 10^−5^	6.10 × 10^−5^
	ORAC	1.10 × 10^−5^	1.30 × 10^−5^	-	5.00 × 10^−6^
	FRAP	7.95 × 10^−4^	6.10 × 10^−5^	5.00 × 10^−6^	-
mg TE/g PBE	DPPH	-	2.05 × 10^−4^	5.00 × 10^−5^	8.00 × 10^−6^
(PBE—1% DMSO)	ABTS	2.05 × 10^−4^	-	6.60 × 10^−5^	3.00 × 10^−6^
	ORAC	5.00 × 10^−5^	6.60 × 10^−5^	-	3.10 × 10^−5^
	FRAP	8.00 × 10^−6^	3.00 × 10^−6^	3.10 × 10^−5^	-

Note: Variables were treated as independent samples.

**Table 4:** Similarities and differences between extracts depending on how concentration values are expressed.

		Water	1%DMSO	*p*-Value *	
Extraction yield	mg/mL PBE	2.91 ± 0.02	3.31 ± 0.01	*p* < 0.05	*p* = 1.81 × 10^−3^
	g PBE/100 g PB	5.77 ± 0.04	6.55 ± 0.03	*p* < 0.05	*p* = 1.92 × 10^−3^
TPC	mg GAE/g PBE	397.4 ± 5.7	401.2 ± 8.1	*p* > 0.05	*p* = 0.540
	mg GAE/mL PBE	1.155 ± 0.017	1.328 ± 0.027	*p* < 0.001	*p* = 6.83 × 10^−4^
	mg GAE/g PB	22.93 ± 0.33	26.27 ± 0.53	*p* < 0.001	*p* = 7.59 × 10^−4^
TFC	mg CE/g PBE	90.4 ± 2.0	93.3 ± 2.3	*p* > 0.05	*p* = 0.100
	mg CE/mL PBE	0.263 ± 0.006	0.309 ± 0.008	*p* < 0.001	*p* = 7.00 × 10^−5^
	mg CE/g PB	5.21 ± 0.11	6.11 ± 0.15	*p* < 0.001	*p* = 7.90 × 10^−5^
DPPH	mg TE/g PBE	881 ± 55	875 ± 13	*p* > 0.05	*p* = 0.855
	mg TE/mL PBE	2.56 ± 0.16	2.896 ± 0.043	*p* < 0.05	*p* = 2.43 × 10^−2^
	mg TE/g PB	50.8 ± 3.2	57.27 ± 0.86	*p* < 0.05	*p* = 2.70 × 10^−2^
ABTS	mg TE/g PBE	1025 ± 41	1034 ± 17	*p* > 0.05	*p* = 0.743
	mg TE/mL PBE	2.98 ± 0.12	3.422 ± 0.055	*p* < 0.05	*p* = 4.16 × 10^−3^
	mg TE/g PB	59.1 ± 2.3	67.7 ± 1.1	*p* < 0.05	*p* = 4.62 × 10^−3^
ORAC	mg TE/g PBE	3188 ± 137	3227.0 ± 301	*p* > 0.05	*p* = 0.806
	mg TE/mL PBE	9.26 ± 0.40	10.7 ± 1.0	*p* < 0.05	*p* = 4.12 × 10^−2^
	mg TE/g PB	183.9 ± 7.9	211 ± 20	*p* < 0.05	*p* = 4.44 × 10^−2^
FRAP—TE	mg TE/g PBE	581 ± 16	588 ± 11	*p* > 0.05	*p* = 0.530
	mg TE/mL PBE	1.688 ± 0.047	1.948 ± 0.036	*p* < 0.05	*p* = 1.62 × 10^−3^
	mg TE/g PB	33.51 ± 0.93	38.52 ± 0.72	*p* < 0.05	*p* = 1.79 × 10^−3^
FRAP—Fe^2+^	mg Fe^2+^/g PBE	265 ± 12	265.8 ± 2.0	*p* > 0.05	*p* = 0.879
	mg Fe^2+^/mL PBE	0.769 ± 0.036	0.880 ± 0.007	*p* < 0.05	*p* = 6.10 × 10^−3^
	mg Fe^2+^/g PB	15.27 ± 0.71	17.40 ± 0.13	*p* < 0.05	*p* = 6.81 × 10^−3^

* Significant differences (*p* < 0.05) between extracts (100% water and 1% DMSO). TPC—Total Phenolic Content; GAE—Gallic acid equivalent; TFC—Total flavonoids content; CE—Catechin equivalent; DPPH—Free Radical Scavenging Effect; ABTS—Radical Cation Scavenging Effect; ORAC—Oxygen Radical Absorbance Capacity; FRAP—Ferric ion Reducing Antioxidant Potential; TE—Trolox equivalent.

**Table 5:**
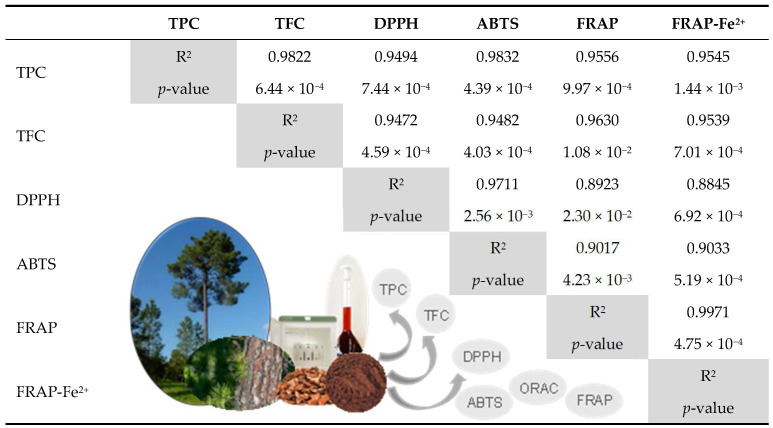
Correlations between the TPC, TFC, DPPH, ABTS, and FRAP values of the extracts—coefficients of determination (R^2^) and *p*-value (*t*-test).

**Table 6:** Identification and quantification of phenolic compounds in water and 1% DMSO extracts from *Pinus pinaster* bark. The results are expressed as phenolic compound (PC) concentration (mg/L PBE, mg/g PBE and mg/kg PB), mean ± SD of three analytical measurements.

Compound		Water	1%DMSO	*p*-Value *	
Cinnamic acid (C_9_H_8_O_2_)	mg PC ^1^/g PBE	1.47 ± 0.10	1.495 ± 0.033	*p* > 0.05	*p* = 0.886
	mg PC/L PBE	4.27 ± 0.30	4.95 ± 0.11	*p* < 0.05	*p* = 2.08 × 10^−2^
	mg PC/Kg PB	84.7 ± 5.9	98.0 ± 2.2	*p* < 0.05	*p* = 2.18 × 10^−2^
Catechin (C_15_H_14_O_6_)	mg PC/g PBE	21.3 ± 1.5	21.77 ± 0.48	*p* > 0.05	*p* = 0.883
	mg PC/L PBE	62.1 ± 4.3	72.1 ± 1.6	*p* < 0.05	*p* = 2.05 × 10^−2^
	mg PC/Kg PB	1232 ± 86	1426 ± 31	*p* < 0.001	*p* = 2.15 × 10^−2^
Gallocatechin (C_15_H_14_O_7_)	mg PC/g PBE	3.89 ± 0.27	3.991 ± 0.088	*p* > 0.05	*p* = 0.841
	mg PC/L PBE	11.31 ± 0.79	13.21 ± 0.29	*p* < 0.05	*p* = 1.7 × 10^−2^
	mg PC/Kg PB	224 ± 16	261.4 ± 5.8	*p* < 0.05	*p* = 1.83 × 10^−2^
Quercetin (C_15_H_10_O_7_)	mg PC/g PBE	2.825 ± 0.085	2.96 ± 0.21	*p* > 0.05	*p* = 0.758
	mg PC/L PBE	8.22 ± 0.25	9.81 ± 0.69	*p* < 0.001	*p* = 1.95 × 10^−2^
	mg PC/Kg PB	163.0 ± 4.9	194.1 ± 4.2	*p* < 0.001	*p* = 2.02 × 10^−2^
Caffeic acid (C_9_H_8_O_4_)	mg PC/g PBE	1.251 ± 0.088	1.320 ± 0.029	*p* > 0.05	*p* = 0.682
	mg PC/L PBE	3.64 ± 0.25	4.370 ± 0.096	*p* < 0.001	*p* = 9.71 × 10^−3^
	mg PC/Kg PB	72.2 ± 5.1	86.5 ± 1.9	*p* < 0.001	*p* = 1.01 × 10^−2^
Taxifolin (C_15_H_12_O_7_)	mg PC/g PBE	18.59 ± 0.93	19.32 ± 0.62	*p* > 0.05	*p* = 0.728
	mg PC/L PBE	54.1 ± 2.7	63.9 ± 2.0	*p* < 0.001	*p* = 7.33 × 10^−3^
	mg PC/Kg PB	1073 ± 54	1265 ± 28	*p* < 0.001	*p* = 7.66 × 10^−3^
Ferulic acid (C_10_H_10_O_4_)	mg PC/g PBE	2.001 ± 0.068	2.009 ± 0.052	*p* > 0.05	*p* = 0.961
	mg PC/L PBE	5.82 ± 0.20	6.65 ± 0.17	*p* < 0.05	*p* = 5.51 × 10^−3^
	mg PC/Kg PB	115.5 ± 3.9	131.6 ± 3.4	*p* < 0.05	*p* = 5.82 × 10^−3^
Ellagic acid (C_14_H_6_O_8_)	mg PC/g PBE	4.69 ± 0.33	4.87 ± 0.11	*p* > 0.05	*p* = 0.780
	mg PC/L PBE	13.66 ± 0.96	16.12 ± 0.35	*p* < 0.05	*p* = 1.39 × 10^−2^
	mg PC/Kg PB	271 ± 19	319.0 ± 7.0	*p* < 0.001	*p* = 1.46 × 10^−2^
Protocatechuic acid (C_7_H_6_O_4_)	mg PC/g PBE	5.92 ± 0.18	5.97 ± 0.34	*p* > 0.05	*p* = 0.942
	mg PC/L PBE	17.22 ± 0.52	19.8 ± 1.1	*p* < 0.05	*p* = 2.35 × 10^−2^
	mg PC/Kg PB	341 ± 10	391.2 ± 8.6	*p* < 0.05	*p* = 2.46 × 10^−2^
o-coumaric acid (C_9_H_8_O_3_)	mg PC/g PBE	1.93 ± 0.14	2.030 ± 0.045	*p* > 0.05	*p* = 0.714
	mg PC/L PBE	5.63 ± 0.39	6.72 ± 0.15	*p* < 0.05	*p* = 1.09 × 10^−2^
	mg PC/Kg PB	111.6 ± 7.8	133.0 ± 2.9	*p* < 0.05	*p* = 1.14 × 10^−2^
Total	mg PC/g PBE	63.91	65.74		
	mg PC/L PBE	185.98	217.59		
	mg PC/Kg PB	3687.7	4305.8		

^1^ PC—Phenolic compound; * Significant differences (*p* < 0.05) between samples (water and 1%DMSO).

## Data Availability

The original contributions presented in this study are included in the article. Further inquiries can be directed to the corresponding author.
